# The Farm Family Exposure Study: Acquavella et al. Respond

**Published:** 2006-11

**Authors:** John Acquavella, Bruce Alexander, Jack Mandel, Christophe Gustin

**Affiliations:** Amgen Inc., Thousand Oaks, California, E-mail: jacquave@amgen.com; University of Minnesota, School of Public Health, Minneapolis, Minnesota; Emory University, Rollins School of Public Health, Atlanta, Georgia; Monsanto Europe S.A., Brussels, Belgium

We thank Mage for his comments. In our article on glyphosate in the Farm Family Exposure Study (FFES) (Acquavella et al. 2004), we used 24-hr urinary creatinine to assess the completeness of daily samples over 5 days for the 48 participating farmers. We erred by summarizing the results as micrograms per deciliter instead of micrograms per day. Using an expected daily excretion of 566 μg/day as the lower end of the normal range (Bingham et al. 1988; Forman 2003), only four 24-hr urine samples over 5 days were below that lower limit. Therefore, the completeness of urine collection for the applicators was exceptional. Further details of the urine collection and our assessment of completeness can be found in a related article (Baker et al. 2005).

Mage criticizes us for not subtracting preapplication urine values in our assessment of systemic dose related to on-study applications. Indeed, seven of the applicators had detectable glyphosate in their urine on the day before their on-study application (Acquavella et al. 2004). Values were 1.1, 2.6, 3.9, 5.3, 8.3, 9.8, and 15.4 ppb. We intentionally did not correct for these initial values for two reasons. First, from an epidemiologic and public health standpoint, it is instructive to know the total dose for farmers during and after an application, which, for example, could then be compared to levels of toxicologic significance. Second, the overestimate caused by this practice is trivial for glyphosate in both an absolute and relative sense. Consider that glyphosate has a U.S. Environmental Protection Agency (EPA) reference dose of 2 mg/kg/day (U.S. EPA 1999), and the highest systemic dose we estimated in our study was 0.004 mg/kg/day. The requested corrections would be to the ten thousandths of a milligram per kilogram per day or less.

Last, Mage calls the fact that we only evaluated one application per farm family a limitation that may vitiate our study. That is a strong indictment for a study that comprehensively assessed exposure for farm families related to a single application of three pesticides to an extent not seen before. We agree that characterizing intraperson variation in absorbed pesticide dose over several seasons would provide valuable information, but that was not the objective of the FFES. Nevertheless, Mage’s claim that we cannot reject the possibility that all 47 applicators have the same exposure distribution is refuted by our observations that absorbed dose was related to specific practices (e.g., not wearing gloves) and by similar findings in the literature that practices dictate absorbed dose (e.g., Arbuckle et al. 2002).

## References

[b1-ehp0114-a0633b] Acquavella JF, Alexander BH, Mandel JS, Gustin C, Baker B, Chapman P (2004). Glyphosate biomonitoring for farmers and their families: results from the Farm Family Exposure Study. Environ Health Perspect.

[b2-ehp0114-a0633b] Arbuckle TE, Burnett R, Cole D, Teschke K, Dosemeci M, Bancej C (2002). Predictors of herbicide exposure in farm applicators. Int Arch Occ Environ Health.

[b3-ehp0114-a0633b] Baker BA, Alexander BH, Mandel JS, Acquavella J, Honeycutt R, Chapman P (2005). Farm Family Exposure Study: Methods and Recruitment Practices for a Biomonitoring Study of Pesticide Exposure. J Expo Anal Environ Epidemiol.

[b4-ehp0114-a0633b] Bingham SA, Williams R, Cole TJ, Price CP, Cummings JH (1988). Reference values for analytes of 24-h urine collections known to be complete. Ann Clin Biochemi.

[b5-ehp0114-a0633b] Foreman J 2003. Clinical presentation of renal disease. In: Rudolph’s Pediatrics (Rudolph C, Rudolph A, eds). New York:McGraw-Hill, 1661.

[b6-ehp0114-a0633b] U.S. EPA (U.S. Environmental Protection Agency) 1999. Glyphosate; Pesticide Tolerance. Final rule. Fed Reg 64(226):66108–66114. Available: http://www.epa.gov/fedrgstr/EPA-PEST/1999/November/Day-24/p30408.htm [accessed 1 October 2006].

